# CIS3/398: Implementation of a Web-Based Electronic Patient Record for Transplant Recipients

**DOI:** 10.2196/jmir.1.suppl1.e8

**Published:** 1999-09-19

**Authors:** L Fritsche, G Lindemann, K Schroeter, A Schlaefer, H-H Neumayer

**Affiliations:** ^1^Department of NephrologyCharité Campus Mitte, BerlinGermany

**Keywords:** Integrated Advanced Information Management Systems, Internet, Electronic Patient Record, Organ Transplantation

## Abstract

**Introduction:**

While the "Electronic patient record" (EPR) is a frequently quoted term in many areas of healthcare, only few working EPR-systems are available so far. To justify their use, EPRs must be able to store and display all kinds of medical information in a reliable, secure, time-saving, user-friendly way at an affordable price. Fields with patients who are attended to by a large number of medical specialists over a prolonged period of time are best suited to demonstrate the potential benefits of an EPR. The aim of our project was to investigate the feasibility of an EPR based solely on "of-the-shelf"-software and Internet-technology in the field of organ transplantation.

**Methods:**

The EPR-system consists of three main elements: Data-storage facilities, a Web-server and a user-interface. Data are stored either in a relational database (Sybase Adaptive 11.5, Sybase Inc., CA) or in case of pictures (JPEG) and files in application formats (e. g. Word-Documents) on a Windows NT 4.0 Server (Microsoft Corp., WA). The entire communication of all data is handled by a Web-server (IIS 4.0, Microsoft) with an Active Server Pages extension. The database is accessed by ActiveX Data Objects via the ODBC-interface. The only software required on the user's computer is the Internet Explorer 4.01 (Microsoft), during the first use of the EPR, the ActiveX HTML Layout Control is automatically added. The user can access the EPR via Local or Wide Area Network or by dial-up connection. If the EPR is accessed from outside the firewall, all communication is encrypted (SSL 3.0, Netscape Comm. Corp., CA).The speed of the EPR-system was tested with 50 repeated measurements of the duration of two key-functions: 1) Display of all lab results for a given day and patient and 2) automatic composition of a letter containing diagnoses, medication, notes and lab results. For the test a 233 MHz Pentium II Processor with 10 Mbit/s Ethernet connection (ping-time below 10 ms) over 2 hubs to the server (400 MHz Pentium II, 256 MB RAM) was used.

**Results:**

So far the EPR-system has been running for eight consecutive months and contains complete records of 673 transplant recipients with an average follow-up of 9.9 (SD :4.9) years and a total of 1.1 million lab values. Instruction to enable new users to perform basic operations took less than two hours in all cases. The average duration of laboratory access was 0.9 (SD:0.5) seconds, the automatic composition of a letter took 6.1 (SD:2.4) seconds. Apart from the database and Windows NT, all other components are available for free. The development of the EPR-system required less than two person-years.

**Conclusion:**

Implementation of an Electronic patient record that meets the requirements of comprehensiveness, reliability, security, speed, user-friendliness and affordability using a combination of "of-the-shelf" software-products can be feasible, if the current state-of-the-art internet technology is applied.

